# Mental health of university students: a cross-sectional study from Qatar

**DOI:** 10.1038/s41598-025-20202-7

**Published:** 2025-10-16

**Authors:** Ghadir Fakhri Al-Jayyousi, Mujahed Shraim, Lily O’Hara, Monica Zolezzi, Noor Al-Wattary, Alla El-Awaisi, Maguy Saffouh El Hajj, Banan Mukhalalati, Hanan Abdul Rahim, Diana Alsayed Hassan

**Affiliations:** 1 Department of Public Health, College of Health Sciences, QU Health, Qatar University, Doha, Qatar; 2 School of Medicine and Dentistry, Griffith University, Gold Coast, Australia; 3 Department of Clinical Pharmacy and Practice, College of Pharmacy, QU Health, University, Doha, Qatar; 4 College of Education and Arts, Lusail University, Doha, Qatar; 5 Vice President for Medical and Health Sciences Office, Health Sector, Qatar University, Doha, Qatar; 6 College of Public Policy, Hamad Bin Khalifa, Doha, Qatar

**Keywords:** Youth, Mental health, Depression, Anxiety, Wellbeing, University

## Abstract

This study explored the prevalence, associated factors, and management strategies related to mental health among university students in Qatar. A cross-sectional survey was conducted among students aged 18 and older. Data were collected on self-reported mental health diagnoses, perceived stress, management strategies, and demographic factors utilizing a self-reported electronic questionnaire. Descriptive, bi-variable, and multivariable logistic regression analyses were performed to identify associations and trends. Among 812 participants (mean age 21.4 years, 84.6% female), 45.5% reported a history of mental illness, with anxiety (38.2%) and depression (27.9%) being most common. A dose–response relationship with life events was observed, whereby students reporting multiple life events had higher odds of a mental illness diagnosis, ranging from OR 2.21 (95% CI 1.40–3.50) for two life events to OR 5.11 (95% CI 2.10–12.42) for five events or more. Despite this burden, only 7.6% of the participants reported that they were seeing a counselor at the time of the survey. The findings reveal a concerning prevalence of mental health conditions among university students in Qatar, particularly anxiety and depression and highlight the urgent need for strategies promoting resilience and mental wellbeing to improve students’ mental health and academic success.

## Introduction

As per the United Nations, youth is defined as “the period between the ages of 15 and 24 years”^1^. This period is marked by substantial changes as youth transition from their restricted role of adolescence to the development of their unique identities as adults^2^. During this period, youth undergo psychological, emotional, biological, and physical changes while shaping their characters^3^. Understanding the mental health and wellbeing of adolescents and young people is essential for the development of health promotion programs to improve their health and wellbeing.

Mental health is a broad term that can be defined as the combination of mental wellbeing, mental health problems, mental illness, and mental health disorders^4^. Mental wellbeing is a positive state that includes concepts such as emotional and psychological resilience, self-awareness, effective stress management, positive interpersonal relationships, a sense of purpose, and the ability to adapt and navigate life’s challenges. Mental health problems are an inexorable component of people’s lives and may therefore co-exist with mental wellbeing. Mental illness; commonly referred to as mental health conditions such as mood disorders, anxiety/fear related disorders, obsessive–compulsive disorders, eating disorders, schizophrenia-spectrum disorders and psychotic states, is a subset of mental health problems and also a subset of mental disorders, with only minimal overlap with mental wellbeing^4^. This nuanced and intersecting definition helps provide clarity about which aspect of mental health is being addressed. Due to the dominance of the biomedical health paradigm, the term mental health more often refers to mental health problems or mental illness.

A 2022 large scale meta-analysis of 192 epidemiological studies found that mental disorders and illnesses have their peak incidence at age 14.5 years, with up to 60% of individuals being diagnosed with their first mental disorder or illness by the age of 25^5^. The World Mental Health Survey conducted by the World Health Organization (WHO) found that 20% of university students reported having a mental health disorder in the past 12 months^6^.

Traditionally, students in the region tend to address mental health related issues, such as stress and burnout, independently, with limited attention given to this topic. One of these studies, reported a high percentage of Qatar University (QU) students having stigmatizing knowledge, attitudes, and beliefs about mental illness, linked to poor mental health literacy with the majority preferring family and friends as treatment and support options^7^. Another study among medical students at QU exploring the prevalence of mental health issues and associated stigma, revealed a prevalence of severe depression (4.4%) and anxiety (10.4%). More than one third of students reported psychological distress (39.6%) and high mental health stigma (31.9%). These findings associated significantly with various sociodemographic factors, such as gender, nationality, parental education and academic progress^8^.

The undergraduate years at university are the time when many risk-taking behaviors for health, such as substance abuse, self-harm, and engagement in violent acts begin^9^ while graduate students may face unique and professional pressures related to research, limited social support, economic precarity, and other stressors related to university-to-work transitions^10^. A systematic review and meta-analysis by Pozuelo et al.^11^ suggested that adolescents with depression have an increased risk of being involved in risk taking behaviors. Nurturing young people’s mental wellbeing can empower them to cope with life’s challenges and stresses, control their emotions, and make sound informed decisions reducing the risk of these behaviors^3^. Youth who are mentally healthy can build and sustain strong relationships with their family, colleagues and the society^5^. It is therefore imperative to promote mental wellbeing in youth through programs that create supportive social, community, and educational environments^12^.

### Qatar and Qatar University

Qatar is the first country in the Eastern Mediterranean Region (EMRO) to have all of its municipalities awarded the WHO title of ‘Healthy Cities’ in 2022. QU is the largest and oldest higher education institution and is the main national university in Qatar. QU comprises 12 colleges and enrolled approximately 25,000 students during 2023–2024, with about 80% female, 67% Qatari nationals, and the largest segment of students enrolled in undergraduate programs^13^. QU was recognized as a “Healthy University” by the WHO in 2022. Focusing on students’ capacity building within the university premises by moving beyond diagnosis and treatment toward social, psychological and emotional human functioning is one of the current higher education objectives^14^. The Healthy Campus Framework supports the promotion of healthy behaviors by utilizing a wellbeing-focused approach. The goal of this approach is to set the basis for the development of a strong integrated and multidisciplinary system within the university infrastructure to sustain the health and wellbeing of the college community^14^. As a result, fostering and investing in health promoting university frameworks is essential to encouraging active citizen involvement, promoting community initiatives, strengthening health policies, and creating supportive environments with a greater focus on prevention^15^. In addition, a healthy campus system can assist in the establishment of an embedded culture of wellbeing and best practices for healthy behaviors; therefore, assisting students to feel more connected to an educational environment that provides them with accessible health-promoting services^14,15^.

While a few previous studies assessed mental health among QU students, most focused on specific colleges. There remains a significant gap in evaluating these rates, their associated factors, and their management strategies within a broader university setting for health promotion and within a more comprehensive framework. This study is part of a larger community assessment project that aimed to assess the health and wellbeing, assets, and needs of QU students from the perspective of students to inform the development of evidence-based multi-strategic health promotion programs that address priority issues and thereby improve students’ health and wellbeing and reduce health inequities^16^. While this study focuses on a national university in Qatar, many of the mental health challenges faced by students such as anxiety, depression, stigma, and stress are shared by young people globally.

The objectives of this study were to describe the prevalence of mental health problems and illnesses, including anxiety and depression and the associated factors with reporting a diagnosis of a mental illness among QU students; describe how students manage their mental health, and describe students’ perceptions of their stress level and their ability to manage stress.

## Methods

### Study design

A cross-sectional study was conducted involving a self-administered online questionnaire completed by QU students. The questionnaire was sent to all students (18 years old and older) with an active registration for Spring and Fall 2022 semesters at QU.

### Sample size

One of the study objectives was to assess the prevalence of mental health problems and illnesses. Assuming the prevalence of any of the outcome measures is 50% (worst-case scenario) then a sample size of 384 students was required based on the following formula: n = [Z^2^ × P × (1 − P)]/d^2^, where Z is the value from standard normal distribution corresponding to desired confidence level (i.e., 1.96 for 95% CI), P is expected proportion and d is the desired level precision (i.e., 0.05 or 5%)^17^.

### Data collection

Data collection started as soon as the research study received approval from Qatar University Institutional Review Board (QU-IRB 1649-EA/21), which approved all protocols. All methods and protocols were performed in accordance with the relevant guidelines and regulations of the Declaration of Helsinki. Data were collected anonymously using an online self-administered questionnaire available in English and Arabic. A questionnaire link was created using the Blue survey platform and mass emails were sent to all actively enrolled QU students through their university email addresses (N = 24,988). The questionnaire link was also shared on QU social media channels and with faculty members to post on their course platforms. A promotion video was recorded by a group of health students and shared on QU social media to promote the survey.

In the email, students were provided with a description of the study, researchers’ information, and contact details. Students were asked for their informed consent to participate in the study. The consent form emphasized the voluntary nature of the study, participants’ anonymity, the confidentiality of information collected, and participants’ right to discontinue at any point of the study with no consequences and risks associated with their participation. Informed consent was obtained from all participants who were then provided access to the online questionnaire.

### Questionnaire

The literature on youth health and wellbeing, college health, and health promotion programs related to college health was reviewed to identify tools, surveys, and variables. The questionnaire consisted of 6 sections: sociodemographic characteristics, health status, mental health, nutrition, physical activity, oral health, health literacy, and health care utilization. The questions for this study on mental health were adapted from the Boynton Health: College Student Health Survey-Questionnaire 2021, the University of Minnesota^18^. These measures were originally developed and validated among university students^16,18^. Only the results from the demographic items and mental health related items are reported in this paper.

Mental health related items in the questionnaire asked participants how many days in the past month were considered “not good” mental health days, if they have been diagnosed with a range of mental illnesses, if they have taken medication for mental illnesses, and if they were currently or in the past six months have seen a mental health counselor/therapist. Participants were asked about the health services they usually access for non-emergency and emergency mental health problems or illnesses, and how frequently they use the hospital Emergency Department (ED) for mental health problems or mental illnesses. Participants were asked about their perceived level of stress and their perceived ability to manage stress on a scale of 1 to 10, with 10 being the highest level of stress and the highest ability to manage stress. Participants were also asked about the total number of life events they experienced over the last 12 months^18^. Life events included getting married, failing a class, experiencing serious physical illness of someone close, death of someone close, being diagnosed as having a serious physical illness, being diagnosed as having a mental illness, spouse/partner conflict, termination of a friendship, being put on academic probation, being fired or laid off from a job, experiencing roommate conflict, family conflict, and lack of health care coverage.

The questionnaire was translated to Arabic by a research group consisting of QU public health faculty members and public health graduate students. Arabic and English versions of the questionnaire were pilot tested in a public health class of 16 undergraduate students to evaluate the clarity and consistency of understanding of each item.

### Data analysis

Descriptive analysis produced summary statistics for means and standard deviations, or median and interquartile range (IQR), for the continuous variables age, number of poor mental health days, perception of stress level, and perception of stress management. Percentage frequencies were calculated for the categorical variables of diagnosis of mental illness, number of life events, medication use, and use of health services. Bi-variable and multivariable logistic regression were used to analyze relationships between self-reporting a mental illness diagnosis (outcome variable) and sociodemographic, psychosocial, and academic-related characteristics as potential predictive variables. The predictor variables included in the models are shown in tables (3–5). Odds ratios (OR) and 95% confidence interval (CI) were used as measures of association. To analyze the relationship between perceived stress levels, perceived ability to manage stress and other factors, we used multiple linear regression analysis with bootstrap (replications[1000], seed[1000], dots [1]). Data were analyzed using Stata (version 18)^19^, and multicollinearity was assessed using variance inflation factors in the regression analysis. Statistical significance was determined using two-sided p-values, with a significance level set at < 0.05.

## Results

### Participant characteristics

A total of 812 students participated in the study (Table 1). The mean age of participants was 21.4 years (standard deviation (SD) 4.5 years) and the majority of participants were female (84.6%), single (89%), undergraduate (90.6%), living with parents (90.9%), with a family income of 30,000 Qatari Riyals (US$ 8240) per month or above. There was a relatively even mix of Qataris (48%) and other nationalities (52%) and over two-thirds of the students (69.4%) had a grade point average (GPA) of 3 (out of 4) or above. All QU colleges were represented, with the highest proportion of participants from the College of Arts and Sciences (24.5%), Engineering (15.5%), Business and Economics (12.6%) and Health Sciences (12.3%).

**Table 1:** Characteristics of participants.

Variable	Frequency (%)
Age mean (SD) (n = 791)	21.4 (4.5)
Gender (n = 812)	
Male	125 (15.4)
Female	687 (84.6)
College (n = 812)	
Arts and Sciences	199 (24.5)
Health Sciences	100 (12.3)
Medicine	60 (7.4)
Pharmacy	52 (6.4)
Dental Medicine	20 (2.5)
Sharia and Islamic Studies	62 (7.6)
Business and Economics	102 (12.6)
Education	68 (8.4)
Law	23 (2.8)
Engineering	126 (15.5)
Academic program (n = 812)	
Undergraduate	736 (90.6)
Graduate	76 (9.4)
Marital status (n = 810)	
Single	721 (89.0)
Married	82 (10.1)
Divorced	7 (0.9)
Nationality (n = 812)	
Qatari	390 (48.0)
Other nationalities	422 (52.0)
Monthly income (Qatari Riyal)^a^ (n = 795)	
< 10,000 (ref)	155 (19.5)
10,000–19,999	173 (21.8)
20,000–30,000	153 (19.3)
> 30,000	314 (39.5)
Living arrangements (n = 812)	
With parents	738 (90.9)
Own household	41 (5.1)
QU student housing	23 (2.8)
With relative or friend	6 (0.7)
Other	4 (0.5)
GPA (n = 791)	
4	181 (22.9)
3.5	208 (26.3)
3	160 (20.2)
2.5	132 (16.7)
2	58 (7.3)
1.5	31 (3.9)
1	7 (0.9)
0	14 (1.8)

Percentages may not total 100% due to rounding.

^a^QAR = Qatari Riyal; 1 USD = 3.64 QAR.

### Prevalence of mental health problems and illnesses

The median number of days participants reported their mental health as “not good” in the last month was 7 days (IQR = 14). Three-quarters of students (74.6%) reported that their mental health had not been good for 15 days or less, and a quarter (25.4%) reported their mental health had not been good for 16 days or more. More than a quarter (29.6%) of students reported that their academic performance had been affected by mental health issues in the past 12 months (Table 2).

**Table 2:** Prevalence of mental health problems and illnesses.

Variable	Frequency (%)
Number of days mental health “not good” in past month (total = 793)	
Median (IQR)	7 (14.0)
Number of days mental health “not good” in past month (total = 793)	
0	289 (36.4)
1–15 days	455 (57.4))
16–30 days	49 (6.2)
Diagnosed with mental illness in past 12 months (total = 792)	
No	746 (94.2)
Yes	46 (5.8)
Ever diagnosed with a mental illness (total = 778)	
No	424 (54.5)
Yes	354 (45.5)
Number of mental illness diagnosis reported by respondents (total = 778)	
0	424 (54.5)
1	89 (11.4)
2	82 (10.5)
3	64 (8.2)
4	49 (6.3)
5	70 (9.0)
Diagnosis of anxiety (total = 798)	
No	493 (61.8)
Yes	305 (38.2)
Diagnosis of depression (total = 799)	
No	576 (72.1)
Yes	223 (27.9)
Diagnosis of anorexia (total = 798)	
No	710 (89.0)
Yes	88 (11.0)
Diagnosis of bulimia (total = 798)	
No	710 (89.0)
Yes	88 (11.0)
Diagnosis of attention deficit hyperactivity disorder (ADHD) (total = 796)	
No	741 (93.1)
Yes	55 (6.9)
Diagnosis of bipolar disorder (total = 797)	
No	728 (91.3)
Yes	69 (8.7)
Diagnosis of obsessive–compulsive disorder (OCD) (total = 795)	
No	708 (89.1)
Yes	87 (10.9)
Diagnosis of panic disorder (total = 799)	
No	668 (83.6)
Yes	131 (16.4)
Diagnosis of post-traumatic stress syndrome (PTSD) (total = 800)	
No	732 (91.5)
Yes	68 (8.5)
Engaged in binge eating in past 12 months (total = 765)	
No	485 (59.7)
Yes	280 (34.5)
Academic performance affected by mental health issues in past 12 months (total = 802)	
No	376 (46.3)
I have this issue but academic performance not affected	186 (22.9)
I have this issue and academic performance has been affected	240 (29.6)

Around one in twenty students (5.8%) reported being diagnosed with a mental illness in the past 12 months. Just under half the students (45.5%) reported having ever been diagnosed with a mental illness, with 11.4% reporting one illness, 10.5% reporting two illnesses, and 23.5% reporting being diagnosed with three or more mental illnesses. The most commonly reported mental illnesses were anxiety (38.2%), depression (27.9%), and panic disorder (16.4%). Eating disorders were prevalent, with 11% of students reporting being diagnosed with anorexia and 11% being diagnosed with bulimia, and more than a third (35.5%) reporting engaging in binge eating in the past 12 months (Table 2).

### Factors associated with students reporting being diagnosed with a mental illness

Nationality and number of life events were independent factors associated with a diagnosis of a mental illness. Non-Qatari students had lower odds of reporting a diagnosis of a mental illness compared to Qatari students (OR 0.60, CI 0.38–0.95). There were no significant associations between reporting a diagnosis of a mental illness and demographic variables of age, academic program, marital status, monthly income, living arrangements, or GPA.

The number of life events was significantly associated with reporting being diagnosed with a mental illness and there was a distinct pattern with the odds increasing at the same rate as the number of life events. Students with two life events had more than double the odds of a diagnosis (OR 2.21; CI 1.40, 3.50), students with three life events had more than triple the odds of a diagnosis (OR 3.47; CI 1.93, 6.26), students with four life events had more than quadruple the odds of a diagnosis (OR 4.34; CI 2.11, 8.91), and students with five life events or more had more than five times higher odds of reporting being diagnosed with a mental illness compared to students reporting no life events (OR 5.11; CI 2.10,12.42) (Table 3).

**Table 3:** Crude and adjusted associations between characteristics of students and reporting being diagnosed with a mental illness.

Variable	Diagnosis of mental illness		Crude association			Adjusted association		
	(Yes) Frequency (%)*	(No) Frequency (%)*	OR (95% CI)	SE	*P*-value	OR (95% CI)	SE	*P*-value
Age–mean (Sd)	21.7 (5.0)	21.3 (4.2)	1.02 (0.99, 1.05)	0.02	0.211	1.02 (0.97, 1.07)	0.02	0.476
Gender								
Male (ref)	46 (39.0)	72 (61.0)						
Female	308 (46.7)	352 (53.3)	1.37 (0.92, 2.04)	0.28	0.124	1.18 (0.732 1.96)	0.3	0.511
Academic program								
Undergraduate (ref)	323 (45.9)	381 (54.1)						
Graduate	31 (41.9)	43 (58.1)	0.85 (0.52, 1.38)	0.21	0.513	0.69 (0.36, 1.34)	0.23	0.274
Marital status								
Single (ref)	310 (44.4)	388 (55.6)						
Married	43 (55.1)	35 (44.9)	1.54 (0.96, 2.46)	0.37	0.073	1.10 (0.56, 2.17)	0.38	0.773
Nationality								
Qatari (ref)	201 (53.7)	173 (46.3)						
Other nationalities	153 (37.9)	251 (62.1)	0.52 (0.39, 0.70)	0.08	< 0.001	0.60 (0.38, 0.95)	0.14	0.028
Monthly income (Riyal)								
< 10,000 (ref)	56 (38.4)	90 (61.6)	1.32 (0.85, 2.08)	0.31	0.218	1.43 (0.86, 2.38)	0.37	0.169
10,000–19,999	76 (45.2)	92 (54.8)	1.48 (0.93, 2.36)	0.35	0.101	1.26 (0.72, 2.21)	0.36	0.416
20,000–30,000	69 (47.9)	75 (52.1)	1.51 (1.01, 2.26)	0.31	0.043	1.19 (0.69, 2.07)	0.33	0.535
> 30,000	147 (48.5)	156 (51.5)						
Living arrangements								
With parents (ref)	317 (44.9)	389 (55.1)	1.66 (0.87, 3.16)	0.55	0.123	1.71 (0.71, 4.09)	0.76	0.23
Own household	23 (57.5)	17 (42.50)	0.85 (0.36, 2.01)	0.37	0.711	1.02 (0.38, 2.74)	0.52	0.962
QU student housing	9 (40.9)	13 (50.1)	1.23 (0.35, 4.28)	0.78	0.748	1.03 (0.25, 4.25)	0.75	0.963
Other	5 (50.0)	5 (50.0)						
Number of life events								
0 (ref)	91 (34.0)	177 (66.0)	1.46 (1.00, 2.14)	0.28	0.049	1.46 (0.96, 2.21)	0.31	0.073
1	85 (42.9)	113 (57.1)	1.95 (1.29, 2.92)	0.4	0.001	2.21 (1.40, 3.50)	0.52	0.001
2	75 (50.0)	75 (50.0)	2.85 (1.68, 4.84)	0.77	< 0.001	3.47 (1.93, 6.26)	1.04	< 0.001
3	44 (59.5)	30 (40.5)	3.90 (1.99, 7.60)	1.33	< 0.001	4.34 (2.11, 8.91)	1.59	< 0.001
4	30 (66.7)	15 (33.3)	4.53 (2.00, 10.31)	1.9	< 0.001	5.11 (2.10, 12.42)	2.32	< 0.001
> 4	21 (70.0)	9 (30.0)						
Academic performance (GPA)								
4.0 (ref)	61 (34.9)	114 (65.1)	1.31 (0.86, 1.99)	0.28	0.208	1.29 (0.80, 2.07)	0.31	0.298
3.5	82 (41.2)	117 (58.8)	1.75 (1.22, 2.73)	0.4	0.013	1.32 (0.79, 2.21)	0.35	0.289
3	74 (48.4)	79 (51.6)	1.81 (1.14, 2.89)	0.43	0.012	0.95 (0.54, 1.66)	0.27	0.859
2.5	63 (49.2)	65 (50.8)	2.16 (1.17, 3.97)	0.67	0.014	1.06 (0.52, 2.16)	0.38	0.868
2	30 (53.6)	26 (46.4)	4.11 (2.07, 8.16)	1.44	< 0.001	1.88 (0.87, 4.07)	0.74	0.107
≤ 1.50	33 (68.8)	15 (31.2)						

In regression analysis, due to small numbers, “Living with relative or friend” category was grouped under “other” category, “Divorce” category was grouped under “single” category, and GPA categories ≤ 1.50 were grouped under “≤ 1.50” category.

*Percentages may not total 100% due to rounding. OR: odds ratio; CI: confidence interval; SE: standard error; ref: reference category.

### Anxiety and depression

Given that anxiety and depression were the most commonly reported mental illnesses that students had been diagnosed with, we explored the factors associated with these two illnesses specifically.

Descriptive analysis revealed that over a third of students (38%) reported a diagnosis of anxiety, with higher rates among female students (39.47%) than male students (31.45%), among married students (43.21%) than unmarried students (37.62%), and among Qatari students (45.6%) compared to non-Qatari students (31.4%). With regards to depression, over a quarter of students (28%) reported a diagnosis of depression, with slightly higher rates in males (29%) than females (28%), among married students (32%) than unmarried students (27%), and among Qatari (34%) compared to non-Qatari students.

In the logistic regression analysis, the association between reporting life events and a history of anxiety diagnosis was significant, following a “dose–response” pattern. The OR for reporting a history of anxiety diagnosis ranged from 1.64 (CI 1.07, 2.52) for students reporting one life event to 5.77 (CI 2.50, 13.29) for those reporting more than four life events, compared to students reporting no life events. Students with a GPA of 3.5 were approximately 2.3 times more likely to report a diagnosis of anxiety compared to students with a GPA of 1 (OR 2.31; CI 1.09, 4.90). The analysis also revealed that non-Qatari students had 45% lower odds of reporting an anxiety diagnosis compared to Qatari students (OR 0.55; CI 0.35, 0.88): (Table 4).

**Table 4:** Crude and adjusted associations between characteristics of students and reporting being diagnosed with anxiety disorder (n = 732).

Variable	Diagnosis of anxiety status		Crude association			Adjusted association		
	Yes (n = 278) Frequency (%)*	No (n = 454) Frequency (%)*	OR (95% CI)	SE	*P*-value	OR (95% CI)	SE	*P*-value
Age–mean (Sd)	21.7 (4.9)	21.4 (4.4)	1.02 (0.98, 1.05)	0.02	0.364	1.02 (0.97, 1.06)	0.02	0.476
Gender								
Male (ref)	38 (32.2)	80 (67.8)						
Female	240 (39.1)	374 (60.9)	1.35 (0.89, 2.05)	0.29	0.159	1.25 (0.76, 2.08)	0.32	0.383
Academic program								
Undergraduate (ref)	258 (38.9)	406 (61.1)						
Graduate	20 (29.4)	48 (70.6)	0.66 (0.38, 1.13)	0.18	0.129	0.68 (0.35, 1.33)	0.23	0.264
Marital status								
Single (ref)	244 (37.3)	410 (62.7)						
Married	34 (43.6)	44 (56.4)	1.30 (0.81, 2.09)	0.31	0.281	0.94 (0.48, 1.83)	0.32	0.85
Nationality								
Qatari (ref)	156 (46.0)	183 (54.0)						
Other nationalities	122 (31.0)	271 (69.0)	0.53 (0.39, 0.71)	0.08	< 0.001	0.55 (0.35, 0.88)	0.13	0.012
Monthly income (Riyal)								
< 10,000 (ref)	47 (32.6)	97 (67.4)						
10,000–19,999	65 (39.4)	100 (60.6)	1.34 (0.84, 2.14)	0.32	0.218	1.41 (0.84, 2.37)	0.37	0.189
20,000–30,000	53 (39.0)	83 (61.0)	1.32 (0.81, 2.15)	0.33	0.27	1.03 (0.59, 1.82)	0.3	0.911
> 30,000	113 (39.4)	174 (60.6)	1.34 (0.88, 2.04)	0.29	0.173	0.92 (0.52, 1.60)	0.26	0.758
Living arrangements								
With parents (ref)	252 (37.8)	414 (62.2)						
Own household	17 (48.6)	18 (51.4)	1.55 (0.79, 3.07)	0.54	0.123	1.65 (0.70, 3.92)	0.73	0.255
QU student housing	6 (28.6)	15 (71.4)	0.66 (0.25, 1.72)	0.32	0.391	0.97 (0.35, 2.69)	0.51	0.96
Other	3 (30.0)	7 (70.0)	0.70 (0.18, 2.75)	0.49	0.614	0.60 (0.13, 2.77)	0.47	0.512
Number of life events								
0 (ref)	62 (25.3)	183 (74.7)						
1	70 (36.3)	123 (63.7)	1.68 (1.11, 2.53)	0.35	0.013	1.64 (1.07, 2.53)	0.36	0.023
2	56 (40.6)	82 (59.4)	2.02 (1.29, 3.15)	0.46	0.002	2.14 (1.33, 3.43)	0.52	0.002
3	40 (53.3)	35 (46.7)	3.37 (1.97, 5.77)	0.92	< 0.001	3.49 (1.95, 6.25)	1.04	< 0.001
4	29 (60.4)	19 (39.6)	4.51 (2.36, 8.60)	1.49	< 0.001	4.97 (2.50, 9.88)	1.74	< 0.001
> 4	21 (63.6)	12 (36.4)	5.17 (2.40, 11.11)	2.02	< 0.001	5.77 (2.50, 13.29)	2.46	< 0.001
GPA								
4.0 (ref)	44 (27.3)	177 (72.7)						
3.5	69 (35.2)	127 (64.8)	1.44 (0.92, 2.27)	0.33	0.112	1.50 (0.92, 2.45)	0.38	0.105
3	60 (41.1)	86 (58.9)	1.86 (1.15, 2.99)	0.45	0.011	1.53 (0.90, 2.59)	0.41	0.113
2.5	48 (39.3)	74 (60.7)	1.72 (1.04, 2.85)	0.44	0.033	0.92 (0.52, 1.65)	0.27	0.785
2	24 (42.9)	32 (57.1)	1.99 (1.05, 3.75)	0.64	0.032	1.09 (0.53, 2.24)	0.4	0.813
≤ 1.50	33 (64.7)	18 (35.3)	4.88 (2.49, 9.53)	1.67	< 0.001	2.31 (1.09, 4.90)	0.89	0.028

*Percentages may not total 100% due to rounding. OR: odds ratio; CI: confidence interval; SE: standard error; ref: reference category; GPA, grade point average. Notes: In regression analysis, due to small numbers, “Living with relative or friend” category was grouped under “other” category, “Divorce” category was grouped under “single” category, and GPA categories ≤ 1.50 were grouped under “ ≤1.50” category.

The logistic regression analysis revealed a statistically significant association between reporting a history of depression and nationality as non-Qatari students had lower odds of reporting a diagnosis of depression compared to Qatari students (OR 0.56; CI 0.34, 0.94). The relationship between reporting life events and a history of depression diagnosis was also significant, demonstrating a “dose–response” relationship. The OR for a history of depression diagnosis increased from 1.68 (CI 1.02, 2.76) among students reporting one life event to 12.56 (CI 5.25, 30.06) among those reporting more than four life events, compared to students who reported no life events (Table 5).

**Table 5:** Crude and adjusted associations between characteristics of students and reporting being diagnosed with depression (n = 733).

	Diagnosis of depression status		Crude association			Adjusted association		
Variable	Yes (n = 207) Frequency (%)*	No (n = 526) Frequency (%)*	OR (95% CI)	SE	*P*-value	OR (95% CI)	SE	*P*-value
Age–mean (Sd)	21.9 (4.8)	21.4 (4.5)	1.02 (0.99, 1.06)	0.02	0.163	1.02 (0.98, 1.08)	0.03	0.292
Gender								
Male (ref)	35 (29.4)	84 (70.6)						
Female	172 (28.0)	442 (72.0)	0.93 (0.61, 1.44)	0.21	0.756	0.87 (0.51, 1.49)	0.24	0.606
Academic program								
Undergraduate (ref)	191 (28.7)	474 (71.3)						
Graduate	16 (23.5)	52 (76.5)	0.76 (0.43, 1.37)	0.23	0.366	0.78 (0.37, 1.65)	0.3	0.521
Marital status								
Single (ref)	181 (27.6)	474 (72.4)						
Married	26 (33.3	52 (66.7)	1.31 (0.79, 2.16)	0.33	0.292	0.74 (0.35, 1.54)	0.28	0.417
Nationality								
Qatari (ref)	117 (34.5)	222 (65.5)						
Other nationalities	90 (22.8	304 (77.2)	0.56 (0.41, 0.78)	0.09	0.001	0.56 (0.34, 0.94)	0.15	0.027
Monthly income (Riyal)								
< 10,000 (ref)	35 (24.3)	109 (75.7)						
10,000–19,999	49 (29.7)	116 (70.3)	1.32 (0.79, 2.18)	0.34	0.289	1.54 (0.87, 2.72)	0.45	0.138
20,000–30,000	36 (26.5)	100 (73.5)	1.12 (0.65, 1.92)	0.31	0.677	0.93 (0.49, 1.76)	0.3	0.825
> 30,000	87 (30.2)	201 (69.8)	1.35 (0.85, 2.13)	0.31	0.2	1.13 (0.61, 2.10)	0.36	0.687
Living arrangements								
With parents (ref)	186 (27.9)	481 (72.1)						
Own household	14 (40.0)	21 (60.0)	1.72 (0.86, 3.46)	0.61	0.126	2.07 (0.83, 5.15)	0.96	0.116
QU student housing	3 (14.3)	18 (85.7)	0.43 (0.13, 1.48)	0.27	0.181	0.61 (0.16, 2.24)	0.4	0.454
Other	4 (40.0)	6 (60.0)	1.72 (0.48, 6.18)	1.12	0.403	1.37 (0.31, 6.07)	1.04	0.682
Number of life events								
0 (ref)	38 (15.5)	207 (84.5)						
1	45 (23.4)	147 (76.6)	1.67 (1.03, 2.70)	0.41	0.037	1.68 (1.02, 2.76)	0.43	0.042
2	43 (30.9)	96 (69.1)	2.44 (1.48, 4.02)	0.62	0.001	2.81 (1.65, 4.77)	0.76	0.001
3	30 (40.0)	45 (60.0)	3.63 (2.04, 6.47)	1.07	< 0.001	4.19 (2.23, 7.89)	1.35	< 0.001
4	29 (59.2)	20 (40.8)	7.90 (4.06, 15.38)	2.69	< 0.001	9.31 (4.55, 19.04)	3.4	< 0.001
> 4	22 (66.7)	11 (33.3)	10.89 (4.88, 24.30)	4.46	< 0.001	12.56 (5.25, 30.06)	5.59	< 0.001
GPA								
4.0 (ref)	38 (23.6)	123 (76.4)						
3.5	41 (20.9)	155 (79.1)	0.86 (0.52, 1.41)	0.22	0.543	0.87 (0.51, 1.51)	0.24	0.633
3	43 (29.7)	102 (70.3)	1.36 (0.82, 2.27)	0.35	0.232	1.07 (0.60, 1.91)	0.31	0.811
2.5	41 (33.6)	81 (66.4)	1.64 (0.97, 2.76)	0.44	0.064	0.76 (0.41, 1.41)	0.24	0.382
2	20 (35.1)	37 (64.9)	1.75 (0.91, 3.37)	0.58	0.094	0.84 (0.39, 1.81)	0.33	0.665
≤ 1.50	24 (46.2)	28 (53.9)	2.77 (1.44, 5.34)	0.93	0.002	1.11 (0.51, 2.43)	0.44	0.785

*Percentages may not total 100% due to rounding. OR: odds ratio; CI: confidence interval; SE: standard error; ref: reference category; GPA, grade point average. Notes: In regression analysis, due to small numbers, “Living with relative or friend” category was grouped under “other” category, “Divorce” category was grouped under “single” category, and GPA categories ≤ 1.50 were grouped under “ ≤ 1.50” category.

### Management of mental health

In this study, 14.5% (111) of students reported receiving psychological counselling in the past six months, 7.6% (61) reported currently seeing a mental health counselor or therapist, 7.2% (58) were currently taking medication, 9.4% visited the Emergency Department (ED) for a mental health issue in the past 12 months, and 31.5% (241) indicated that they would need psychological counselling in the future (Table 6).

**Table 6:** Management of mental health.

Variable	Frequency (%)
All students currently taking medication for any mental illness (total = 803)	
No	744 (92.8)
Yes	58 (7.2)
Students with a mental health diagnosis currently taking medication for any mental illness (total = 352)	
No	308 (87.5)
Yes	44 (12.5)
All students currently seeing a mental health counselor or therapist (total = 803)	
No	742 (92.4)
Yes	61 (7.6)
Students with a mental health diagnosis currently seeing a mental health counselor or therapist (total = 354)	
No	303 (85.6)
Yes	51 (14.4)
All students receiving psychological counselling in past 6 months (total = 764)	
No	412 (53.9)
No but will need in future	241 (31.5)
Yes	111 (14.5)
All students who visited the ED for a mental health issue in the past 12 months (total = 763)	
No	691 (90.6)
Yes	72 (9.4)
Students with a mental health diagnosis who visited the ED for a mental health issue in the past 12 months (total = 328)	
No	285 (86.9)
Yes	43 (13.1)
All students: number of times visited the ED in the past 12 months (total = 643)	
0	763 (58.5)
1	97 (15.1)
2	79 (12.3)
3	36 (5.6)
4	13 (2.0)
≥ 5	42 (6.5)
Students with a mental health diagnosis: number of times visited the ED in the past 12 months (total = 272)	
0	134 (49.3)
1	47 (17.3)
2	49 (18.0)
3	14 (5.2)
4	7 (2.6)
≥ 5	21 (7.6)
All students: level of ease or difficulty in finding information about stress and mental health conditions (total = 770)	
Don’t know	86 (11.2)
Very difficult	104 (13.5)
Fairly difficult	192 (24.9)
Fairly easy	209 (27.1)
Very easy	179 (23.3)
All students: level of ease or difficulty in finding out about activities good for mental wellbeing, such as meditation, exercise, and walking (total = 769)	
Don’t know	49 (6.4)
Very difficult	25 (3.3)
Fairly difficult	97 (12.6)
Fairly easy	303 (39.4)
Very easy	295 (38.4)

Although half the students (388, 50.4%) reported that they found it fairly or very easy to find information on how to manage stress and mental health conditions, more than a third (296, 38.4%) reported finding it fairly or very difficult to find such information. More than three-quarters of the students (598, 77.8%) found it fairly or very easy to find out about activities that are good for mental health and wellbeing, including meditation, exercise, and walking. However, 15.7% of students (122) found it fairly or very difficult to find out about such activities.

Among students with a diagnosis of a mental illness, 12.5% (44) reported currently taking medication, 14.4% (51) reported currently seeing a mental health counselor or therapist, and 13.1% (43) reported visiting the emergency department (ED) for a mental health issue in the past 12 months. Of these, 17.3% (47) of these students visited the ED once, 18% (49) twice, and 15.4% (52) reported three or more visits.

### Perceptions of stress levels and ability to manage stress

On a scale from one to ten, with ten being the most stressed, the mean perceived level of stress over the past 30 days was 6.1 (SD 6.8). On a scale from one to ten, with ten being the most effective, the mean perceived ability to manage stress over the past 30 days was 5.7 (SD 2.5). Given the temporal proximity to the COVID-19 pandemic, we also asked students how the COVID-19 pandemic had affected their day-to-day stress levels. Responses were almost equally divided between no change (369, 46%) and increased stress (359, 44.8%), though around 10% of students (74) felt that the COVID-19 pandemic had decreased their stress levels.

We explored the relationship between perceived level of stress and ability to manage stress and having a diagnosis of a mental illness, taking medication for any mental illness, and seeing a mental health counselor or therapist. Students diagnosed with a mental illness had a higher perceived level of stress by 0.8 (CI 0.4, 1.2) and a lower perceived ability to manage stress by 0.4 (CI 0.01, 0.7) compared to students without a diagnosis of a mental illness (Table 7). Students on medication for a mental illness had a higher perceived level of stress by 0.1 (CI − 0.6, 0.8) and a lower perceived ability to manage stress by 0.8 (CI 0.2, 1.4) compared to students not on medication. Students seeing a mental health counselor or therapist for a mental illness had a higher perceived level of stress by 0.5 (CI − 0.2, 1.3) and a lower perceived ability to manage stress by 0.9 (CI 0.2, 1.5) compared to students not seeing a counselor or therapist.

**Table 7:** The relationship between mental health characteristics of students and perceived levels of stress and perceived ability to manage stress.

	Perceived level of stress	Perceived ability to manage stress
Any mental health condition diagnosis		
No (n = 354)	5.8 (2.7), 6 (4–8)	5.9 (2.6), 6 (4–8)
Yes (n = 423)	6.5 (2.5), 7 (5–8)	5.5 (2.3), 5 (4–7)
Taking medication for any mental health condition		
No (N = 743)	6.1 (2.6), 6 (4–8)	5.8 (2.5), 6 (4–8)
Yes (N = 58)	6.2 (2.7), 7 (4–8)	5.0 (2.3), 5 (3–7)
Students seeing mental health counselor or therapist		
No (N = 741)	6.1 (2.6), 6 (4–8)	5.8 (2.4), 6 (4–8)
Yes (N = 61)	6.6 (2.7), 7 (5–8)	4.9 (2.5), 5 (3–7)

The data values represent mean (standard deviation), median (interquartile range).

## Discussion

This study focused on three of the four components of mental health as defined by Granlund et al.^4^: mental wellbeing, mental health problems, and mental illness. The findings of our study suggest that the prevalence of mental health problems and illnesses among this sample of students is significant. Several studies highlighted an incremental trend in mental health disorders as the result of the recent global COVID-19 pandemic, and have highlighted that younger adults have been particularly affected^20–22^. The finding from our study that almost half the students (mean age 21.4 years) had been diagnosed with a mental illness is consistent with the large scale meta-analysis that found that 60% of people had been diagnosed with a mental illness by the age of 25 years^5^. These figures are considerably higher than the Australian study that found that 25% of students had been diagnosed with a mental illness^23^. Our study found that around 6% of students had been diagnosed with a mental illness in the past 12 months which is considerably lower than the finding from the WHO World Mental Health Study that found that 20% of students reported a mental health condition in the past 12 months, though not necessarily a formal diagnosis of a mental illness^6^. This may explain the significant discrepancy between these figures.

During the COVID 19 pandemic, two studies from QU provided insights on the wellbeing of health profession students at Qatar University. The first study explored the prevalence of burnout and its relationship to anxiety and empathy, reporting prevalent occurrence of burnout among the students, with anxiety being a strong predictor for burnout^24^. In the second study, the Coping Reservoir Model served as a theoretical framework to gain a deeper insight into students’ experiences of resilience and burnout during the pandemic. It highlighted the impact of personal, social, and academic challenges on their coping mechanism^25^. These studies called for increasing awareness and developing interventions to enhance student well-being.

The extent of mental health conditions in our study seems to be pronounced with almost one quarter of the students reporting a history of three or more diagnosed mental health conditions. The prevalence rates of anxiety (38.2%) and depression (27.9%) in our study are somewhat similar to another study’s findings also among QU students (34.2%, 32% respectively)^26^, however, lower than the rates found among university students in one university in the UAE (55%, 38%, respectively)^27^ and another study conducted among university students in Saudi Arabia (53%, 54%, respectively).

We did not find a significant difference in the prevalence of mental illness diagnoses among male and female students. This contrasts with the finding by Mahgoub et al.^8^ that male medical students at QU experienced higher rates of self-reported mental health problems than female students. Our study results relating to nationality were in line with those from Mahgoub et al. We found that Qatari students had higher odds of having a mental illness diagnosis in general, and a diagnosis of anxiety or depression specifically, compared to non-Qatari students, while Mahgoub et al.^8^ found that Arab students had higher odds of reporting anxiety than students from other nationalities.

In our study, diagnoses of eating disorders were prevalent, with one in nine students reporting ever being diagnosed with anorexia, one in nine with bulimia, and more than a third reporting engaging in binge eating in the past 12 months. Eating disorders have the highest mortality rate of any mental illness^28^ and the median age of onset for anorexia nervosa, bulimia nervosa, and binge eating disorder is 17 to 22 years^5^. However, there is a lack of research on the prevalence rates of diagnosed eating disorders in the Middle East, and studies have focused on assessing risk of eating disorders rather than rates of diagnosed eating disorders. The measurement indicators are therefore different. Bearing this in mind, it remains useful to compare our findings with other studies. A 2020 review found that 13–55% of Middle East populations studied are at high risk for eating disorders, with the prevalence risk higher among young females^29^. A 2022 scoping review of the prevalence of high risk disordered eating specifically focused on adolescents and young adults in the Middle East found a wide range of prevalence across countries with the highest rates in female (75.8%) and male (69.6%) university students in Egypt^30^. With respect to high-risk for anorexia nervosa, the study found prevalence rates to be highest in Oman (9.5%), which was similar to the prevalence rate of diagnoses in our study (11%), however the rates of high-risk for bulimia (up to 1%) were much lower than the rates in our study (11%) (6). The rates of high-risk for binge eating disorder (up to 1.8%) in the review were also much lower than the prevalence of binge eating in the past 12 months (34.5%)^30^. Given that assessing risk for eating disorders is not the same as assessing rates of eating disorder diagnoses, the scoping review concluded that future research studies need to also include clinical diagnosis to determine the prevalence of diagnosed eating disorders^30^. There is a pressing need to understand the factors that contribute to the onset of eating disorders, and to develop programs to reduce the risk to students at this vulnerable age.

More than a quarter of students in our study indicated that their academic performance had been affected by mental health issues in the past 12 months. Although the question did not specify the direction of the effect, it is reasonable to assume that students interpreted this question as meaning a negative effect. The finding that students felt their academic performance is negatively impacted by mental health issues seems to be intuitive, but contrasts to the findings of a cross sectional study of students in Kurdistan, which found that although there was a strong correlation between academic motivation and mental health, there was no correlation between mental health and academic achievement when assessed using Goldberg’s General Health Questionnaire (GHQ-28), which includes subscales for anxiety, depression, social dysfunction, and somatic symptoms^31^. Further research is required to determine the relationship between mental health, academic motivation, and academic performance.

Our findings reflect that help seeking from a mental health counselor or therapist among university students remains low, despite the growing incidence of mental health conditions in this population. Although counselling and disability support have been regularly provided for university students, studies in Arab countries have indicated that university students are either not aware of the types of mental health services that they provide, or have negative attitudes that foster shame at admitting to having mental health problems, and thus may rather seek social support from family members or friends when faced with mental illness rather than seeking professional help^7,32,33^. Fear of cultural and societal stigma has been extensively reported in studies in the Middle East as important barriers to help-seeking. Although there is significant progress and country-wide campaigns to de-stigmatize mental health, it is likely to take some time to see an actual effect on the society’s believe system^34^.

Several higher education institutions are implementing action plans to address this expected rise in mental health conditions among university students^35,36^. Raising mental health awareness and decreasing stigma is perhaps one of the most adopted strategies at the university level and one that may be able to demonstrate short term effects, particularly regarding negative attitudes towards mental illness and help-seeking behavior. Frameworks designed specifically for university settings may offer guidance on adopting mental health strategies. An example is the Australian University Mental Health Framework, which was developed by student and university stakeholders and experts in the field, and offers recommendations for creating mentally healthy academic environments where students can flourish both academically and personally^37^. The framework also offers recommendations on how the mental health community should partner with universities to improve student mental health and wellbeing. At its core, the framework emphasizes the importance of early intervention, risk reduction, and promotion of mental health across the lifespan. It provides a structured guide for identifying risk factors, assessing needs, and implementing tailored strategies that consider individual strengths and cultural contexts. With a focus on resilience, recovery, and empowerment, the framework underscores the importance of collaboration across disciplines and sectors to create supportive environments and equitable access to quality mental health care. Through its inclusive and person-centered approach, this framework could enhance mental health outcomes and foster a society where mental health is prioritized and stigma is minimized. Another important strategy that has been implemented is training students as mental health mentors or peer helpers who assist fellow students in acknowledging that mental health issues exist, help them to recognize the signs of distress and teach them basic self-care including how and where to go for professional care. One approach to promoting student mental health is training peers to deliver mental health first aid (MHFA)^38^. The MHFA course is an internationally recognized training program that helps to identify, understand and respond to signs of mental illnesses and substance use disorders. This type of training is also becoming increasingly important in healthcare professionals’ education. Strategies like this can not only decrease mental health stigma, but also help students in improving their communication skills and gain confidence in supporting someone in emotional distress.

### Strengths and limitations

This study was the first to assess the prevalence of mental health conditions, associated factors, management and coping strategies among a sample of students at QU, which is the primary national governmental institution of higher education in the State of Qatar. These findings contribute valuable insights to the global discourse on youth mental health within the health-promoting universities context, particularly in regions with similar cultural or academic environments. The inclusive approach in this study, targeting students from all colleges, allowed for more impactful results to be obtained through inferential analysis. Furthermore, the tools used in this study were previously validated and widely used in the literature. Finally, the survey tool in this study was administered to students in both Arabic and English languages to ensure that the language barrier does not influence the response rate or the students’ ability to understand the questions. Nevertheless, the findings of this study should be interpreted with caution due to several limitations. Data collection has been associated with the missing responses for some variables, a limitation inherent to the survey-type research. However, the missing data were accounted for by using valid percentages. Furthermore, the response rate was relatively low, which might be justified by the length of the survey tool. In addition, the survey tool was a self-administered questionnaire; therefore, a possibility of response bias and/or exaggeration of results or missing details might have occurred. Therefore, future research should focus on conducting qualitative research approaches to further understand the perception of students about their mental health conditions and their associated factors. Furthermore, future research can focus on examining the role of university on addressing the mental health needs of students, and on evaluating access, availability, and effectiveness of existing support systems. Moreover, this study relied on self-reported mental health diagnoses, which may be prone to recall bias, misclassification, or misunderstanding of diagnostic terms. Some participants may have reported overlapping conditions (e.g., both bipolar and major depressive disorder) that are not clinically distinct, leading to potential diagnostic overlap. As diagnoses were not clinically verified, findings reflect participants’ perceptions rather than confirmed clinical assessments. Given that the student population at QU is predominantly female (approximately 80%), the prevalence estimates reported in this study may not be generalizable to university populations with different gender distributions. This demographic characteristic should be considered when interpreting the findings and comparing them to other academic settings. Finally, it is recommended for academic institutions, healthcare organizations, and policymakers to collaborate in developing effective strategies that raise awareness among young individuals about the importance of mental health measures and procedures, in order to enhance overall mental wellbeing. These collaborations can lead to the development and implementation of comprehensive strategies, such as accessible mental health services, awareness programs, and supportive campus environments, to promote and prioritize the mental health and wellbeing of university students.

## Conclusion

The results of this study provide a broad insight into the mental health and wellbeing of university students in Qatar; data which is important to guide university-wide strategies that align with QU’s efforts within the scope of its recent recognition by WHO as a “Healthy University”. This data is also meaningful to youth globally within the context of health promoting universities. These efforts have the ultimate aim of incorporating mental and physical health into the university culture, processes and policies which contribute to a broader movement toward an evidence-informed approach to student mental health. Maintaining sustainable health-promoting strategies during higher education is essential in preparing students for future work and professional roles.

## Data Availability

The data sets generated and analyzed during this project will be available from the corresponding author upon reasonable request.
